# Septic thromboembolism of the femoral artery from mitral valve endocarditis

**DOI:** 10.1016/j.jvscit.2025.101781

**Published:** 2025-03-09

**Authors:** Cole E. Ogrydziak, Robert C. McMurray, Anthony Katras

**Affiliations:** a Department of General Surgery, Tripler Army Medical Center, Honolulu, HI; b Department of Vascular Surgery, Tripler Army Medical Center, Honolulu, HI

**Keywords:** Acute limb ischemia, Femoral artery bypass, Infective endocarditis, Mitral valve disease, Open surgical repair, Septic thromboembolism, Staphylococcus epidermidis

## Abstract

Acute limb ischemia (ALI) demands prompt diagnosis and intervention to prevent irreversible tissue damage. Although ALI is more common in older patients, especially due to atrial fibrillation, younger patients with ALI require a broader differential to determine the specific cause. This case report describes a 19-year-old male presenting with ALI from septic thromboembolism due to methicillin-sensitive *Staphylococcus epidermidis* infective endocarditis, and it highlights a few unique management considerations. Specifically, an atypical presentation of ALI should place an infectious etiology higher on the differential, as the presence of infection impacts conduit choice in the event of creating an arterial bypass. Additionally, the vessels involved in the septic thromboembolism can become extremely friable and inflamed, as in this case, to the point that they may not be amenable to an endovascular approach and, even with an open mechanical thrombectomy, still necessitate bypass. Finally, optimal management of this complex case relied on the coordinated efforts of multiple medical specialties, highlighting the importance of a multidisciplinary team approach.

Lower extremity acute limb ischemia (ALI) has an incidence of 1.5 cases per 10,000 persons per year, typically as a result of embolism, thrombosis, dissection, or trauma.^1^ Arterial embolism by and large originates from the left heart, historically as a consequence of rheumatic valvular disease (which has been all but virtually eliminated), but now commonly as a result of atrial fibrillation.^2^^,^^3^ Mortality from ALI due to arterial embolism ranges from 4% to 15%.^3^ Patients are commonly elderly (mean age, 66 years) and male (3:2 ratio).^4^ However, when young patients without typical risk factors present with embolic ALI, less common sources of embolism need to be considered, to include paradoxical emboli, vasculitis, hypercoagulable states, cardiac tumors, and endocarditis.^5^ Here, we report a case of lower extremity ALI in a 19-year-old previously healthy male due to septic thromboembolism from methicillin-sensitive *Staphylococcus epidermidis* (MSSE) infective endocarditis (IE) related to undiagnosed mitral valve prolapse (MVP). There is limited literature specifically addressing the surgical management of septic thromboembolism arising from IE, particularly in young patients. This case report aims to underscore the surgical considerations unique to managing ALI in this specific setting and emphasize the importance of a multidisciplinary approach to care. Details and images were published with the patient’s consent.

## Case report

**Fig 1:**
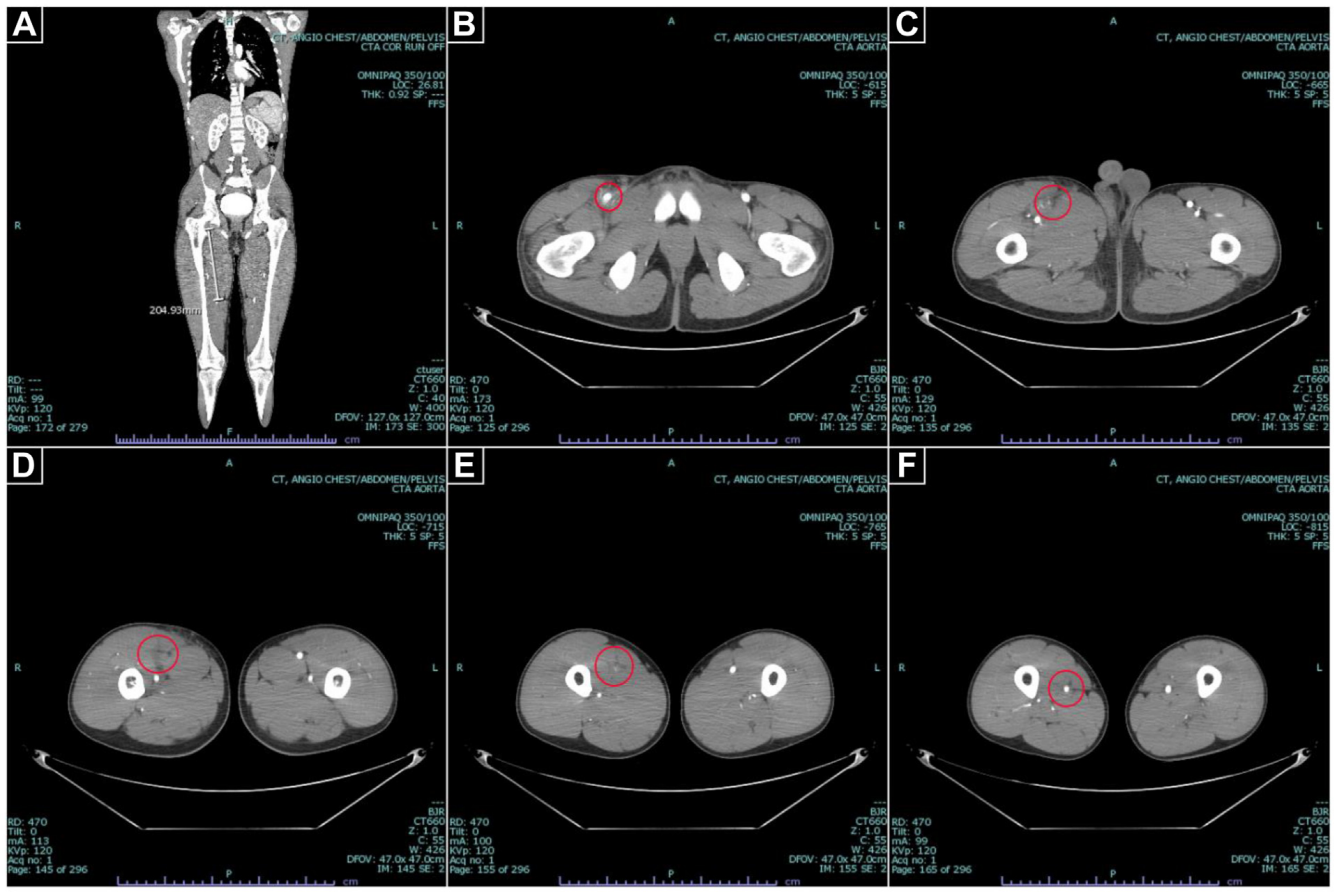
Computed tomography angiography (CTA) of the aorta with runoff. Representative CTA images demonstrate loss of arterial enhancement for an approximately 20-cm segment **(A)** of the right superficial femoral artery from its branch point with the femoris profunda **(B)** and reconstitution distally **(F)** with notable periarterial inflammation of the occluded segment **(C**-**E)**.

A 19-year-old male aboard a naval ship patrolling the tropics developed acute mild right lower quadrant pain with radiation to the right groin, all while at rest. After 1 day of symptoms that progressed to moderately severe pain, at most radiating to the upper thigh, he was evaluated by his ship’s medical specialist and administered acetaminophen and ibuprofen. After 2 days of symptoms progressing to severe pain at rest with radiation to the level of the knee as well as nausea, vomiting, and anorexia, he was medically evacuated to the nearest military treatment facility (MTF) for further evaluation. He arrived at this MTF at 4 days of symptoms, where computed tomography angiography (CTA) revealed a 20-cm segment of occlusion of the right superficial femoral artery (SFA) with notable periarterial inflammation (Fig 1). Systemic anticoagulation was initiated, and he was transferred to next-closest MTF with vascular surgical capabilities. He arrived to our facility on the evening of 5 days of symptoms with normal vital signs, right lower extremity rest pain to the level of the knee, and low-grade leukocytosis. However, he had no sensory or motor losses, and he had audible Doppler signals (ie, Rutherford category I), so he underwent surgery the next morning on symptom day 6.

An open approach was selected as opposed to an endovascular approach primarily due to an unfortunate lack of suction thrombectomy catheters at our facility, but this approach was also considered due to concern for the significant periarterial inflammation noted on imaging. After making a standard longitudinal cutdown to expose the common femoral artery (CFA) at its bifurcation with the profunda femoris (PFA) and superficial femoral artery (SFA), these vessels were indeed notably inflamed. They were ecchymottic and thin-walled in appearance, but without purulence of significant adjacent soft tissue inflammation. Control of the CFA, PFA, and SFA was obtained, after which 5000 units of systemic heparin was administered. We proceeded with thrombectomy using a #5 Fogarty balloon catheter. However, despite gentle passage of the balloon catheter into the SFA, this maneuver perforated the SFA near its bifurcation with the PFA. This arteriotomy was oversewn with 6-0 polypropylene suture and thrombectomy was attempted again, this time returning approximately 20 cm of thrombus with additional sweeps made until no more thrombus was retrieved and brisk back-bleeding was appreciated.

Upon reinspection of the involved vessels, the first 2 to 3 centimeters of both the SFA and the PFA were extremely stenosed and, in fact, appeared obliterated to the point that recannulation was not felt to be feasible even with vasodilatory agents. We decided to reestablish in-line flow using an appropriately sized 8-mm Gore polytetrafluoroethylene (PTFE) interposition graft from the CFA to SFA. This was followed by placement an appropriately sized 6-mm PTFE jump-graft off of this first bypass to the PFA. Subsequent angiogram confirmed in-line flow through the graft and distal vessels, and the patient demonstrated strong multi-phasic distal Doppler signals. Given the unusual inflammation of the vessels, a biopsy of the bypassed SFA was also sent for pathology.

**Fig 2:**
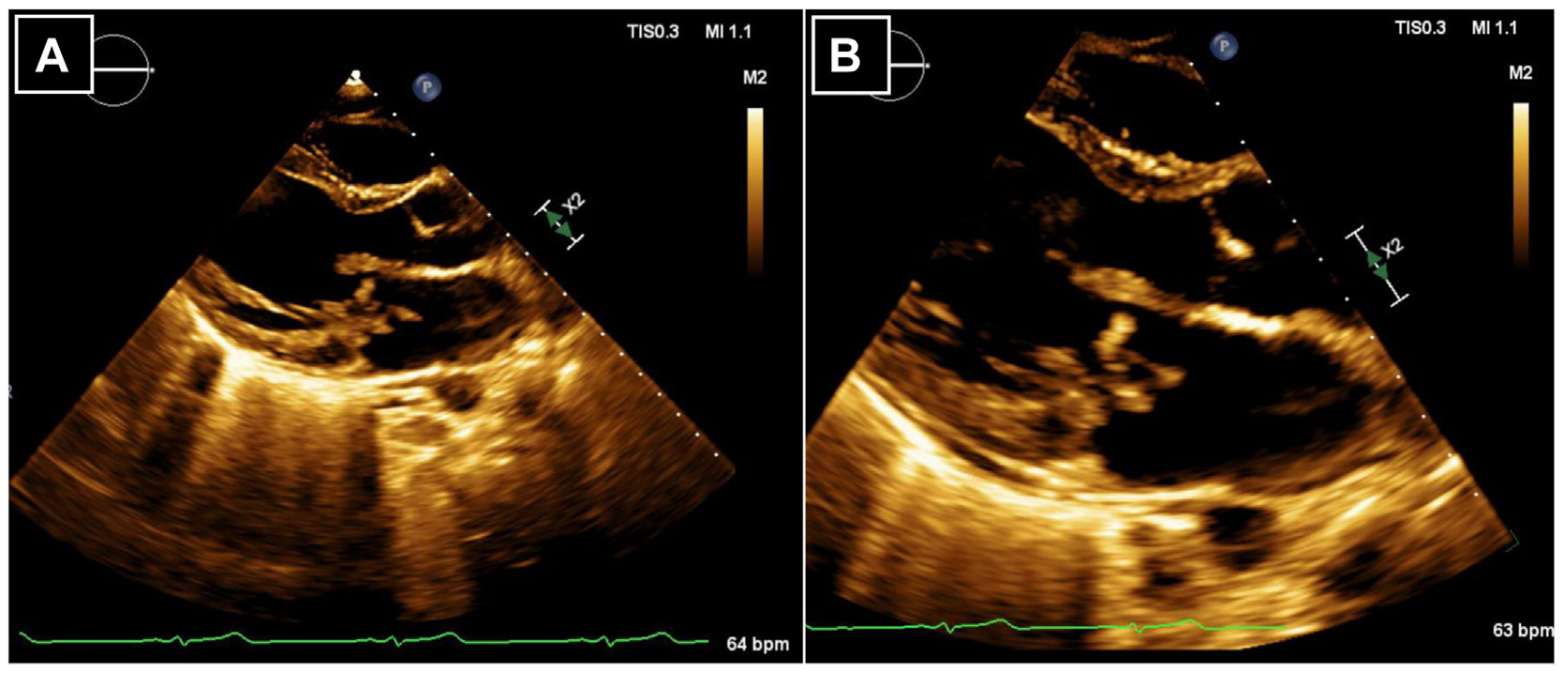
Echocardiogram. Representative images **(A-B)** of the echocardiogram demonstrating a large mobile multilobulated echodensity on the posterior mitral valve leaflet, measuring up to 2.5 centimeters in length.

An extensive workup to determine the etiology of his ALI was carried out, to include inflammatory markers, coagulation assays, antiphospholipid syndrome antibodies, antineutrophil cytoplasmic antibodies antibodies, blood cultures, a hereditary connective tissue disorder panel, a transthoracic echocardiogram (TTE), and specialty consultation with our Rheumatology, Genetics, Cardiology, and Infectious Disease colleagues. One of two blood cultures detected MSSE, which a polymerase chain reaction assay was able to detect within a few hours after blood draw. Although this was initially thought to be a contaminant, TTE and final pathology results suggested otherwise. TTE demonstrated severe mitral valve regurgitation due to MVP with a 2.5-cm mobile and multilobulated vegetation on the posterior mitral valve leaflet concerning for IE (Fig 2). Final pathology of the biopsied SFA segment demonstrated embedded Gram-positive cocci. A specialized 16S bacterial polymerase chain reaction assay was performed on the biopsy specimen that definitively confirmed MSSE. For these findings, on postoperative day 12 from thrombectomy, and after additional cardiothoracic workup, he underwent open replacement with a mechanical mitral valve. The involved leaflet was noted to have several ruptured chords and an area of vegetation and inflammation, but the final pathology of the mitral valve demonstrated only degenerative changes and acute inflammation without any organisms on Gram stain and isolated from subsequent culture.

From the time IE was diagnosed and to include 8 weeks after mitral valve replacement, the patient was continued on suppressive cefazolin for MSSE coverage as well as rifampin to mitigate risk of prosthetic graft biofilm formation. He was last evaluated in our clinic about at 6 weeks postoperatively where he was recovering appropriately, reporting no lower extremity pain. We had planned for a surveillance right lower extremity arterial duplex ultrasound at 1 year postoperatively, but in the interim, the patient had medically retired from the military and returned to his home of record where he now receives care.

## Discussion

Atypical presentations of ALI first and foremost need to seriously consider lesser-common causes, such as IE, because they can be elusive. We suspect the following the sequence of events around the patient’s initial presentation. He described repeat abrasions and cuts to his hands in his military occupational specialty involving mechanical repair work. Additionally, he had a tattoo placed about a few weeks prior to symptom onset. One of these events is felt to be the inciting event, where the patient’s previously undiagnosed MVP is a recognized risk factor for IE. Briefly, MVP is a common and often benign condition, but certain features such as thickened leaflets, redundancy, male sex, and age over 45 years old are associated with an increased risk of complications such as IE.^6^ Moreover, the presentation of IE in patients with MVP can be atypical, such as in this case, potentially delaying diagnosis. In the weeks leading up to initial presentation, the patient endorsed frequent heat intolerance (although there were no documented fevers ≥38 °C) and diaphoresis, but these symptoms were overlooked in the setting of his deployment in the tropics where the climate is tropical and notoriously hot and humid. Likewise, his work aboard the naval ship is also in hot and humid conditions, especially in these engine room areas. He had no cellulitis or active wounds on his hands or tattooed skin. He had no other medical known problems and denied intravenous drug use.

In retrospect, the patient’s presenting symptoms were consistent with IE, but at the time of presentation, they were not compelling, given the aforementioned context. The modified Duke criteria^7^ are supposed to be the cornerstone of IE diagnosis. However, this case illustrates their limitations, namely the time it takes to fulfill the criteria such that they may not be fully apparent at the time of intervention. Prior to surgery, the patient met no major microbiologic, imaging, or surgical criteria; and the only minor criteria he satisfied was the vascular phenomena with the SFA thromboembolism. Upon further workup, he eventually met multiple major microbiologic (microorganisms that occasionally or rarely cause IE isolated from 3 or more separate culture sets) and imaging (echocardiography showing vegetation with new significant valvular regurgitation) criteria and thereby pathologic criteria as well for definite endocarditis. Of course, he had already undergone surgery by then, with our decisions made with the information available to us at the time.

Reasonable arguments for an endovascular vs an open approach could be made in this scenario. We chose open for two main reasons. Practically, we did not have any suction thrombectomy catheters available at the time this patient presented. Apart from this, we suspected that, due to the significant periarterial inflammation noted on CTA, the involved vessels would be friable, posing an increased risk of vessel perforation via endovascular approach. One of the main operative considerations we think this case highlights is that the vessels were so extremely friable that we still made an iatrogenic perforation even with our open approach. The intraoperative findings, including significant vessel friability and inflammation, the iatrogenic arteriotomy despite careful tissue handling, and especially the obliterated SFA and PFA segments necessitating bypass, we feel validate this decision-making. An additional benefit of an open approach in this situation was that the SFA biopsy helped confirm MSSE as the causative organism. Coagulase-negative staphylococci, including MSSE, are common commensals of the skin and are typically associated with contamination, as we initially thought the blood culture results suggested. However, MSSE infections are increasingly recognized as important pathogens in health care-associated infections, particularly in the context of indwelling medical devices, and they can also be seen, although less commonly, in cases associated with intravenous drug use.^8^

The decision to utilize PTFE for the bypass grafts was based on several factors. Although autogenous saphenous vein is generally preferred for below-knee bypasses, the need for a graft that could accommodate the different sizes of the CFA, SFA, and PFA, as well as the desire to minimize operative time by avoiding vein harvest, favored the use of PTFE.^9^ Furthermore, the patient’s young age, good overall health, short bypass segments, and above-knee distal anastomoses suggested favorable patency rate for PTFE grafts, as supported by some studies showing comparable outcomes to vein grafts in similar situations.^10^ Perhaps most importantly, at the time of surgery, infectious etiology was not our primary diagnostic consideration. The leading differential was vasculitis, namely given the patient’s age and lack of obvious systemic symptoms suggestive of infection. Consequently, the potential increased risk of infection associated with prosthetic grafts was not a major factor in our decision-making. In retrospect, given the confirmed diagnosis of IE, an autogenous vein graft would have been the more prudent choice due to its advantages in the presence of infection.

Fortunately, the patient has not experienced any infectious complications, likely aided by the prolonged course of antibiotics administered postoperatively. Specifically, we consulted with our Infectious Disease colleagues immediately once IE was demonstrated by TTE, at which time he was started on vancomycin and ceftriaxone, soon changed to vancomycin and nafcillin, and then with rifampin added to mitigate biofilm formation on the PTFE graft. This therapy was continued up to and after the time of his open mitral valve replacement (which occurred 12 days from embolectomy) until Gram stain and culture results from the mitral valve replacement returned negative. This occurred 23 days from embolectomy (11 from mitral valve replacement), at which time, he was switched to cefazolin 2 grams every 8 hours and rifampin 600 mg orally every evening, and this was continued for 8 weeks after mitral valve replacement. It is important to acknowledge that the patient’s longterm risk of graft infection, although around 1% or less by some estimates, is not zero.^11^

The decision to forgo lower leg fasciotomy, despite an estimated 6 days of symptoms, was based on a few findings. On arrival, the patient presented with viable ALI (ie, Rutherford category I), noted by intact sensorimotor function with audible Doppler signals. This remained stable with frequent neurovascular checks until the time of surgery, and at the conclusion of surgery, angiogram demonstrated in-line flow, and there were strong Doppler signals. Although prolonged ischemia can indeed lead to compartment syndrome, close clinical monitoring and judicious decision-making remain paramount.

Finally, the successful management of this complex case was contingent upon interdisciplinary collaboration. The coordinated efforts of, namely, Vascular Surgery, Infectious Disease, Cardiothoracic Surgery, and Cardiology were crucial in establishing the correct diagnosis and executing the appropriate interventions to include surgery, antibiotic therapy, and long-term anticoagulation.

## Conclusion

This case report details the management of a 19-year-old male presenting with ALI secondary to septic thromboembolism as a rare manifestation of MSSE IE, and with it, we want to emphasize important management considerations unique to this atypical presentation. This experience reinforces the importance of considering infectious etiologies in atypical presentations of ALI, particularly in younger patients, as such situations would favor autologous vein for use in any potential bypass procedures. Considering the extremely friable vessels we encountered that, even with an open approach, ultimately required a bypass, we suggest exercising caution if selecting an endovascular approach, considering an open approach, and at the least being prepared for possible bypass. The complexity of this case necessitated a coordinated approach involving multiple medical specialties, underscoring importance of a multidisciplinary team. Overall, this case adds valuable insights to the limited existing literature on the management of septic thromboembolism and will hopefully stimulate further discussion on this clinical entity.

## Funding

None.

## Disclosures

None. Additionally, the views expressed are those of the author and do not reflect the official policy or position of the Department of the Army, Defense Health Agency, Department of Defense, or the United States Government.
